# Biological composition analysis of a natural medicine, Faeces Vespertilionis, with complex sources using DNA metabarcoding

**DOI:** 10.1038/s41598-021-04387-1

**Published:** 2022-01-10

**Authors:** Xiaoying Zhang, Wenxiu Wang, Xiaolei Yu, Yuxia Liu, Wenhui Li, Hongxia Yang, Ying Cui, Xiaoxuan Tian

**Affiliations:** State Key Laboratory of Component-Based Chinese Medicine, Tianjin University of Traditional Chinese Medicine, Tianjin, 301617 China

**Keywords:** Bioinformatics, Molecular biology, Medical research

## Abstract

Faeces Vespertilionis is a commonly used fecal traditional Chinese medicine. Traditionally, it is identified relying only on morphological characters. This poses a serious challenge to the composition analysis accuracy of this complex biological mixture. Thus, for quality control purposes, an accurate and effective method should be provided for taxonomic identification of Faeces Vespertilionis. In this study, 26 samples of Faeces Vespertilionis from ten provinces in China were tested using DNA metabarcoding. Seven operational taxonomic units (OTUs) were detected as belonging to bats. Among them, *Hipposideros armiger* (Hodgson, 1835) and *Rhinolophus ferrumequinum* (Schober and Grimmberger, 1997) were the main host sources of Faeces Vespertilionis samples, with average relative abundances of 59.3% and 24.1%, respectively. Biodiversity analysis showed that Diptera and Lepidoptera were the most frequently consumed insects. At the species level, 19 taxa were clearly identified. Overall, our study used DNA metabarcoding to analyze the biological composition of Faeces Vespertilionis, which provides a new idea for the quality control of this special traditional Chinese medicine.

## Introduction

Traditional Chinese medicine is one of the oldest systems of traditional medicine, which originated more than 2500 years ago. Since the publication of the Yellow Emperor’s Classic of Internal Medicine (“Huangdi Neijing” in Chinese) in the Han dynasty^1^, many classical medical books and pharmacopeia in China have contained the prescriptions and clinical application of medicines with complex sources^2,3^. Some medicines, such as Faeces Vespertilionis, Faeces Trogopterori^4^ (the dry faeces of *Trogopterus xanthipes* Milne-Edwards, 1867), Faeces Leporum, Silkworm Faeces^5^ (the dry faeces of *Bombyx mori* Linnaeus, 1758), and *Cordyceps*^6^ (a complex mixture of *Cordyceps sinensis* (BerK.) Sacc. and larvae of the family Hepialidae), have been used currently. Faeces Vespertilionis is the dried excrement of bats, which can be collected in the wild all year round as a commercial medicinal material. After removing the sediment, removing impurities, and drying, Faeces Vespertilionis is usually used to improve eyesight, promote blood circulation, promote digestion and alleviate food retention in clinical practice^3,7–9^.

Although some authors have argued that the diversity of hosts and prey might affect Faeces Vespertilionis quality and efficacy^2,10,11^, the species involved in this medicine have not been precisely restricted by pharmacopeia or other practical criteria thus far. In appearance, Faeces Vespertilionis has been described as oblong, slightly pointed at both ends, easy to break, and able to be crumbled into small particles or powder^2^. All of these characteristics are shared by most bat faeces. Although previous studies have shown that approximately 100 species, 24 genera and 6 families of Chiroptera distributed in China could be taken as the origin of Faeces Vespertilionis, there has been no survey of the actual sources of this fecal traditional Chinese medicine^12^. It is worth noting that bats are hosts to a broad range of different viruses, therein also comprising a reservoir of potential zoonotic pathogens. In particular, the spread of coronavirus disease 2019 (COVID-19) has aroused people’s attention to the hosts of Faeces Vespertilionis^13^. On the other hand, the diet of bats has been regarded as the direct source of medicinal effects of Faeces Vespertilionis in previous reports^2,10,11^. These bats mainly feed on nocturnal flying insects^2^ belonging to Lepidoptera, Diptera, Hymenoptera and Coleoptera. The variety of insects makes it hard to guarantee the uniform quality of this traditional Chinese medicine.

Unfortunately, traditional means cannot easily provide species-specific identification for this kind of complicated natural medicine^12^. Broken debris of insects, such as appendages, eyes, setae and thorns^11^, could be observed by microscope. Nevertheless, identifying hosts or other biological composition at the species level is impracticable. Moreover, no species-specific compounds of Faeces Vespertilionis have yet been reported. In the natural medicine identification field, DNA barcoding has emerged as a new molecular tool for species identification^14^. Furthermore, DNA barcoding technology is able to identify traditional medicines with complex species sources^15^. Therefore, there is an urgent need to develop novel diagnostic tools to solve this problem.

DNA metabarcoding has revolutionized species identification methods^16^. This method is based on the high-throughput sequencing (HTS) of DNA barcode regions, amplified using universal polymerase chain reaction (PCR) primers^17^. Currently, HTS technologies can generate millions of sequences concurrently. Therefore, DNA metabarcoding enables us to quickly characterize a very large number of species present in an environmental sample in a single experiment^16,18,19^. Dietary analyses have been facilitated by the advent of metabarcoding and its application to the analysis of faeces or stomach contents^20^, especially for the analysis of the diet of insectivorous bats^21,22^. It is feasible that the biological composition of Faeces Vespertilionis could be discovered by metabarcoding, regardless of whether diversity is from the diet or species introduced due to collection, storage or transportation.

To our knowledge, this report is the first application of metabarcoding to depict the biological composition of a natural medicine with complex sources. Twenty-six Faeces Vespertilionis samples from ten provinces in China were selected, and the taxa involved were explored. We aimed to identify the bat hosts and other biological compositions of Faeces Vespertilionis, which should be an innovation for the quality control of this kind of fecal medicines.

## Results

### Sequencing results and data filtering

The Illumina sequencing of 26 PCR products generated a total of 17,794,946 paired-end reads. A total of 12,175,994 reads were obtained after filtering out low-quality reads, trimming tags and primers, and merging the paired-end reads. After retaining 232 bp sequences, dereplicating reads and removing chimeras, unique sequences were clustered at a 98% similarity threshold. Then, operational taxonomic units (OTUs) with a relative abundance greater than 0.01% of total reads of the 26 samples were retained. Finally, 243 OTUs and 4,758,016 reads remained. Rarefaction curves analysis indicated that the number of sequences and sequencing depth were sufficient for this study (Fig. S1).

### Taxonomic identification of bats

**Figure 1:**
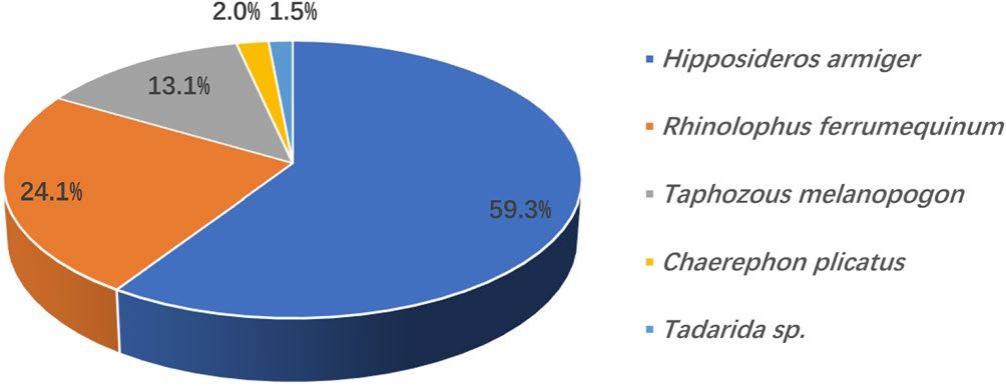
Taxonomic information and average relative abundance of bat species in the samples.

**Figure 2:**
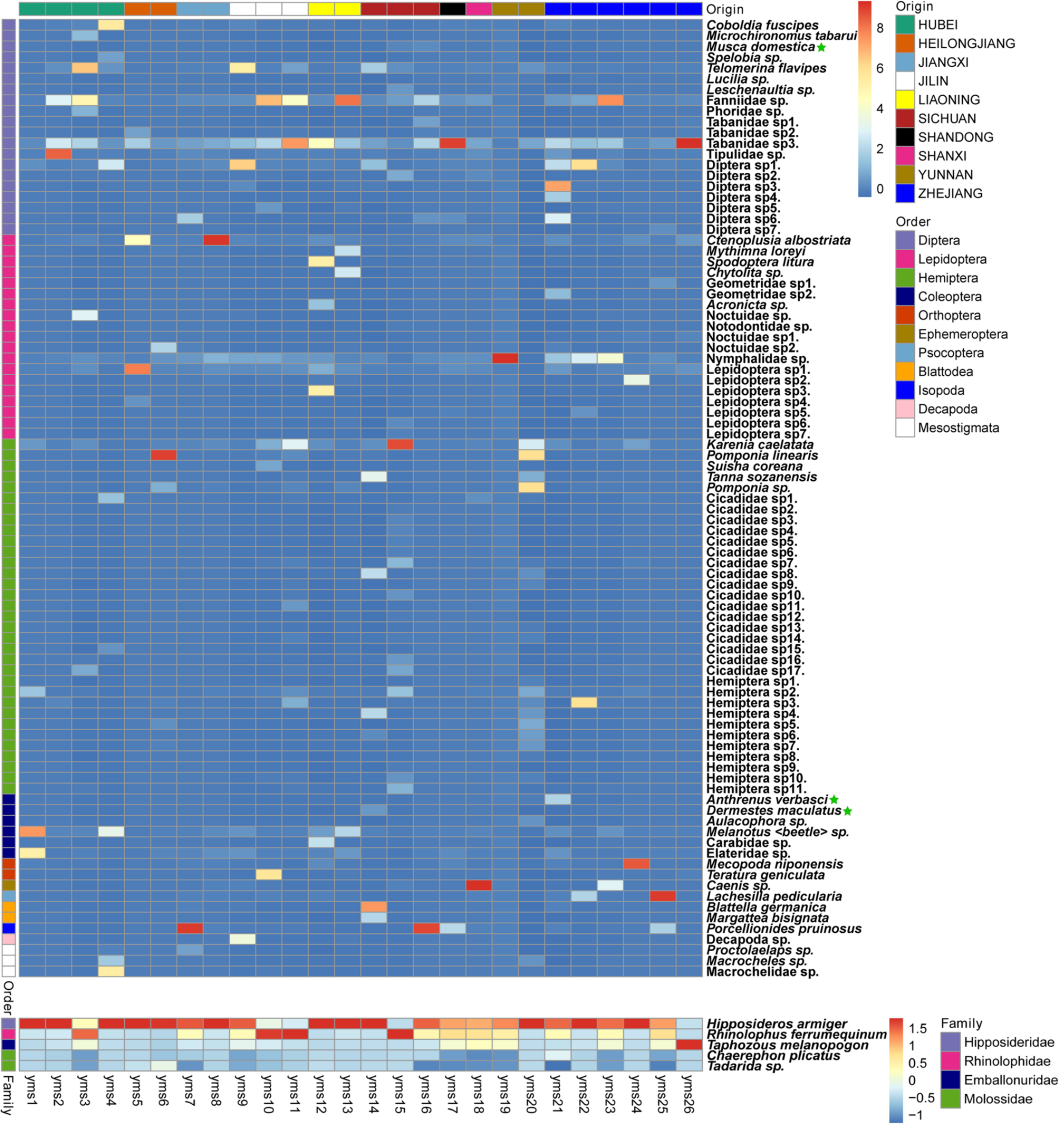
Detection of species in each Faeces Vespertilionis sample. Heatmap was plotted using the package pheatmap (v1.0.12) in R (v4.1.0). The upper part reflects the biological composition in the samples of Faeces Vespertilionis. Species were categorized at the order level, and samples were grouped by sample origin. The asterisks indicate that the species might have been introduced due to hygiene problems. Moreover, the lower part reflects the composition of bat species in each sample. Species were categorized at the family level.

We identified five bat species comprising 1,037,606 reads (Table S1). Among them, the average relative abundance (i.e., the total relative abundance of each taxon across all samples divided by 26) of *H. armiger* accounted for 59.3% of the 26 Faeces Vespertilionis samples, followed by *R. ferrumequinum* (24.1%) (Fig. 1). Two or more bat species could be identified in each sample, with *H. armiger* and *R. ferrumequinum* being the main host types within most collected samples in this study (Fig. 2).

### Biodiversity analysis

**Figure 3:**
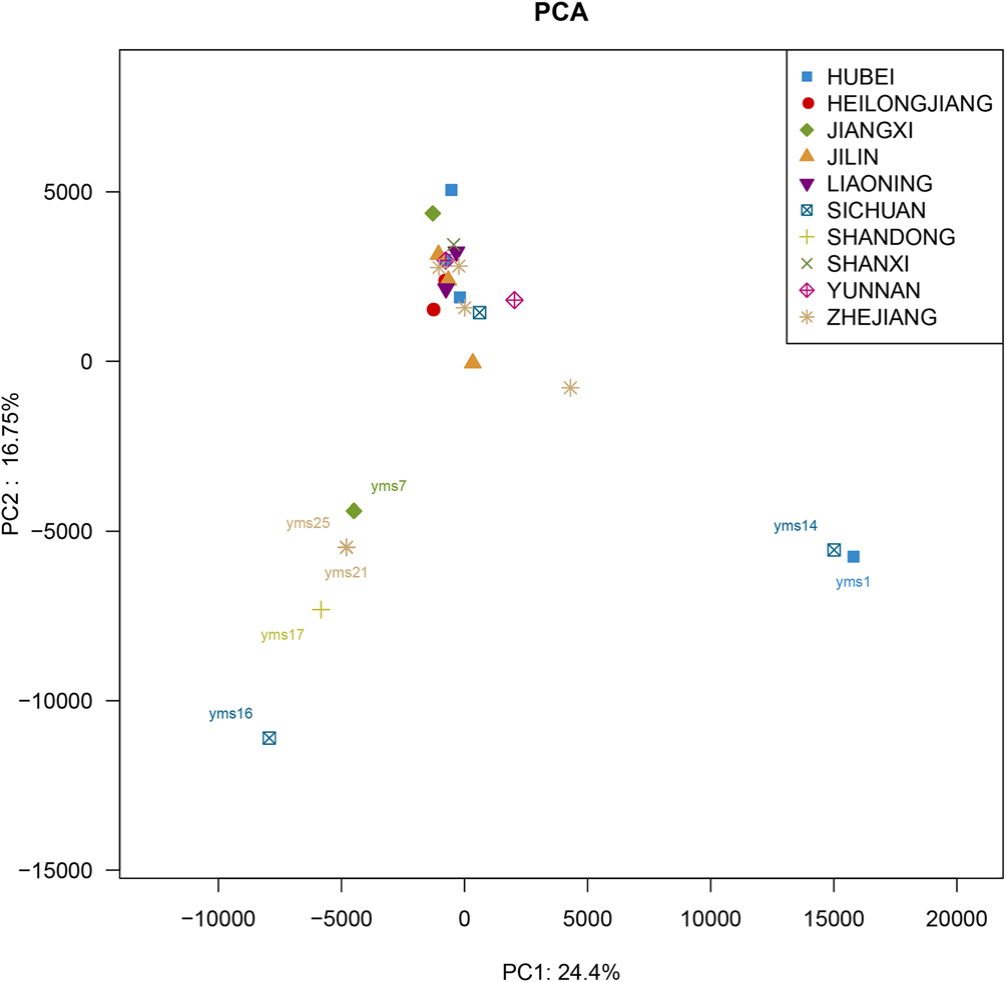
PCA. The default parameters of R (v2.12.1) were used for PCA and data visualization. Points of different colors or shapes represent samples of Faeces Vespertilionis from different origins.

Principal component analysis (PCA)^23^ and non-metric multidimensional scaling (NMDS)^24^ were utilized to calculate beta diversity and to analyze the differences between samples (Fig. 3 and Fig. S2). The PCA results showed that the samples were clustered into three groups. The first was composed of yms1 from HUBEI Province and yms14 from SICHUAN Province. The second group comprised yms7 from JIANGXI Province, yms16 from SICHUAN Province, yms17 from SHANGDONG Province, yms21 from ZHEJIANG Province, and yms25 from ZHEJIANG Province. The remaining samples formed the third group. Both the PCA and NMDS results implied that the origin was not the main explanation for the biodiversity variation between samples.

**Figure 4:**
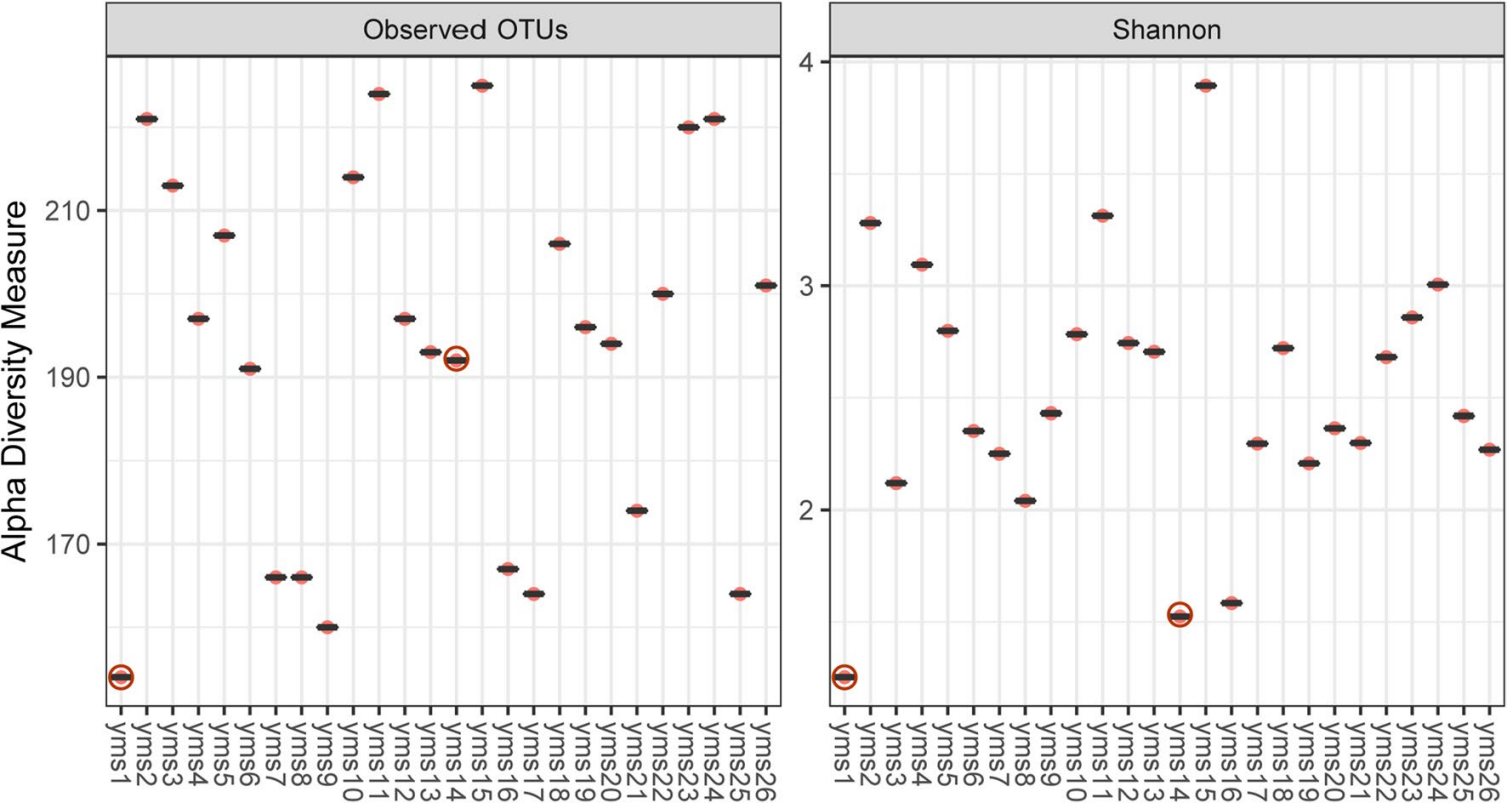
Alpha diversity analysis within each sample. The phyloseq (v1.36.0) and ggplot2 (v3.3.3) packages in R (v4.1.0) were used for alpha diversity analysis and data visualization. The red circles highlight that samples are different from others.

The alpha diversity^25^ of each sample, calculated with observed OTUs, and the Shannon entropy index were used to evaluate the biodiversity within samples. The observed OTUs constituted the number of different OTUs observed within an individual sample. The Shannon entropy index indicated the richness and evenness of the species present. As shown in Fig. 4, approximately 150–230 OTUs belonging to invertebrates could be observed in every 10 g of sample. Although the number of OTUs observed varied from sample to sample, the distribution of Shannon entropy was mainly between 2 and 3 when considering OTU evenness. It is worth noting that although samples yms1 and yms14 had relatively low diversity, the former had fewer OTUs, and the latter presented a more unbalanced species distribution. Both indices were useful to assess the complexity of Faeces Vespertilionis.

### Taxonomic identification of biological composition

**Figure 5:**
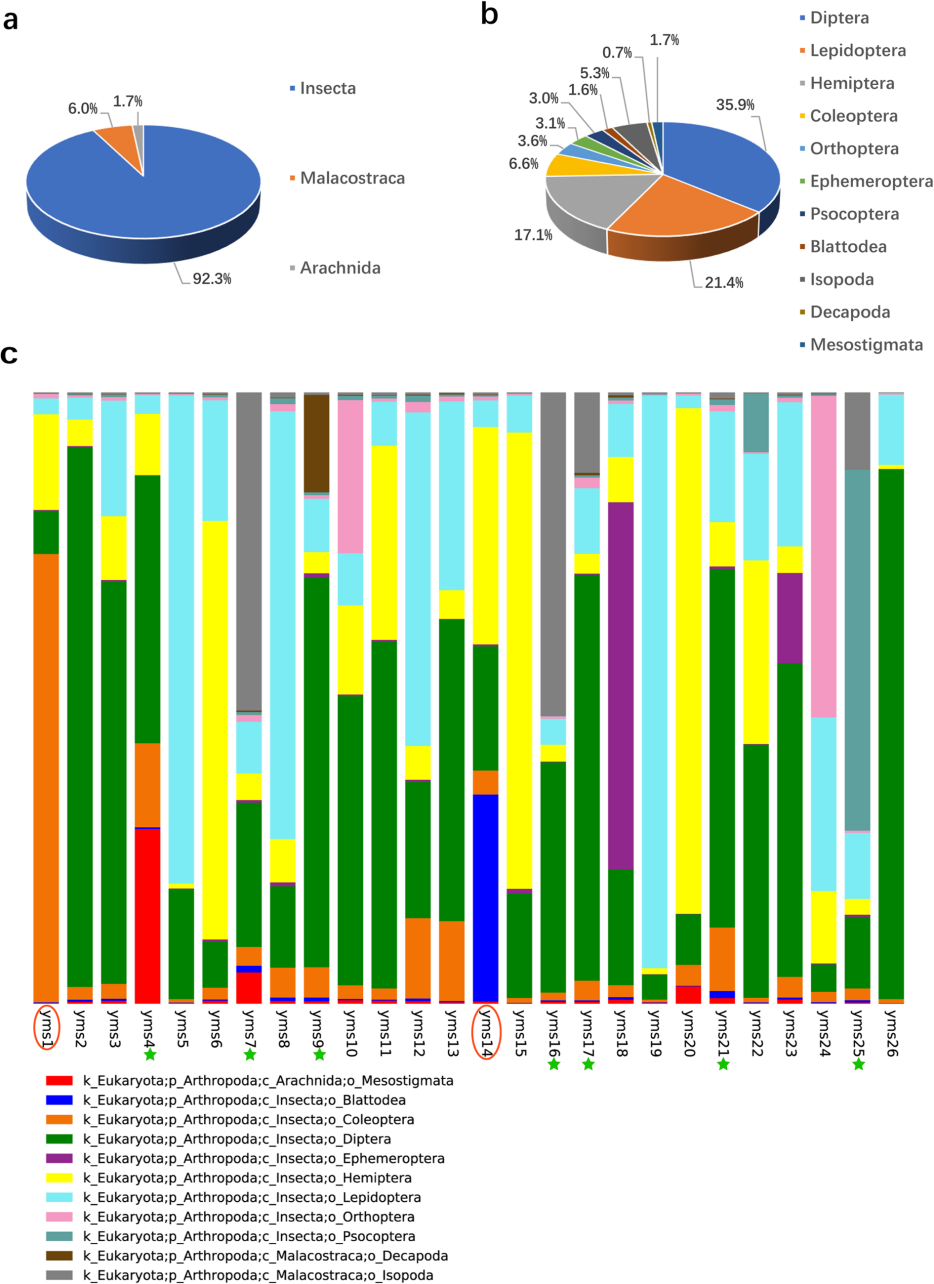
Taxonomic composition analysis. (**a**) Taxa at the class level. (**b**) Taxa at the order level. (**c**) Taxonomic composition analysis at the order level in each sample. The columns of different colors represent different taxa, and the lengths of the columns represent the relative abundances of the taxa. The red circles highlight that samples are different from others, and the asterisks indicate the samples containing non-Insecta taxa.

In further evaluation of biological composition, 94 OTUs were identified at the order level. Among them, 68 were further identified at the family level and assigned to 27 taxa. Twenty-four were identified at the species level and assigned to 19 taxa (Table S2). The OTUs identified were divided into three classes (Fig. 5a) and 11 orders (Fig. 5b), of which 89 OTUs with 543,376 reads belonged to Insecta. The result was clearly dominated by Diptera OTUs. Lepidoptera was the second most abundant, followed by Hemiptera. Several taxa within Coleoptera, Orthoptera, Ephemeroptera, Psocoptera and Blattodea were also recorded at a much lower frequency (Fig. 5b). In contrast to most samples, Coleoptera accounted for the largest proportion in yms1, while Blattodea accounted for a larger proportion in yms14 (Fig. 5c).

Tabanidae sp3. represented a significant proportion of Diptera OTUs, while Nymphalidae sp. was representative of Lepidoptera (Fig. 2—upper panel). In addition, several Cicadidae OTUs were also detected, and Cicadae Periostracum is a traditional Chinese medicine traditionally has been used to improve eyesight and treat colds with fever^2^.

Arachnida (11,222 reads) and Malacostraca (13,263 reads) species were also detected. We identified Arachnida in yms4 and Malacostraca in yms7, yms9, yms16, yms17, yms21, and yms25 (Figs. 2, 5c). The Arachnida OTUs included 3 mites that are often predatory on insects or nematodes (*Proctolaelaps* sp., *Macrocheles sp.*, and Macrochelidae sp.). The cosmopolitan woodlouse *Porcellionides pruinosus* (Brandt, 1833) and *Decapoda* sp. corresponded to the Malacostraca OTUs.

## Discussion

As hypothesized, DNA metabarcoding was a powerful method for host identification. This is the first report that describes two bat species (*H. armiger* and *R. ferrumequinum*) as the main Faeces Vespertilionis hosts. *H. armiger*^26^ and *R. ferrumequinum*^27^ live in groups in caves, abandoned tunnels, roofs or abandoned houses. They are widely distributed in China and mainly prey on nocturnal flying insects, such as Diptera and Lepidoptera.

Our approach provided a detailed biological composition of the samples. At the order level, our results were congruent with previous studies, since Diptera and Lepidoptera were frequently observed in fecal samples^28,29^. In fact, these two orders constituted the majority of samples in the whole study, regardless of read counts or OTU number. The non-Insecta OTUs, first reported by us, suggests that a broader range of taxa within Faeces Vespertilionis can be identified using DNA metabarcoding than by traditional identification methods. The three mites parasitizing insects or nematodes discovered also indicate the sensitivity of this method. It is noteworthy that the presence of taxa with bioactivity (e.g., Cicadae Periostracum) that we detected might be fundamental to Faeces Vespertilionis efficacy.

To ensure species-level taxonomy assignment accuracy, the following aspects were considered. First, our study utilized a portion of the mitochondrial cytochrome c oxidase subunit I (COI) “Folmer” region. This region is a widely applied animal barcode^30,31^, especially for the identification of bat species and their diet^21,32^. Second, since the balance between fidelity and amplification success rate must be considered for polymerase selection during biological composition analysis experiments, Tks Gflex DNA Polymerase, which has been successfully used in similar studies, was adopted in our research^33,34^. Finally, the bioinformatics processing parameters we used have been adopted by previous related studies^31,35,36^. In addition, we manually checked the taxonomic assignment results in MEGAN and investigated the geographical distribution and life history of taxa to ensure reliable species-level identification. Additionally, although we evaluated our selected primers through NCBI Primer-BLAST and found that the primers could amplify different Chiroptera families and different Insecta orders, metabarcoding markers (including the COI “Folmer” region) may have skewed our results due to the existence of primer bias^37,38^.

The stability of traditional medicines benefits from controlling the biological composition within them^39^. However, the origins of Faeces Vespertilionis samples are not related to bat species or other biological compositions in their faeces, which suggests that the quality of this medical material could not be controlled by only limiting its sources. However, from the perspective of diversity, the samples were indeed clustered into three groups, which might be related to the presence of other orders from Insecta, such as Coleoptera and Blattodea, and might also be related to non-Insecta taxa. Regarding the abundant biodiversity of the samples, the sample size should be increased in the future to find key factors affecting the stability of the biological level of the samples to control the biodiversity and ensure the stability and uniformity of medicine quality.

Our results shed light on the biological composition of this commercial medicinal material, in which both dietary species and introduced species due to hygiene problems were observed. In our study, *Ctenoplusia albostriata* (Bremer & Grey, 1853)^40^ from Lepidoptera was found to be distributed in all samples from ten provinces in China. *Coboldia fuscipes* (Meigen, 1830)^41^, an oyster mushroom fly from Diptera, was also found to be distributed in all samples from ten provinces in China. Additionally, the housefly *Musca domestica* (Linnaeus, 1758)^42,43^, the carpet beetle *Anthrenus verbasci* (Linnaeus, 1767)^44^, and the carrion beetle *Dermestes maculatus* (De Geer, 1774)^45^ were detected in our samples, which might have been introduced due to hygiene problems.

In summary, exemplified by Faeces Vespertilionis, we have shown that the DNA metabarcoding approach is a practical solution to explore the species composition of traditional medicines with highly complex components. The quality control of natural medicines might benefit from our explorative research.

## Methods

### Samples and DNA extraction

In this experiment, we collected 26 samples of Faeces Vespertilionis from ten provinces in China (Table 1). Approximately 10 g of each sample was lysed with 80 ml of 1.5% sodium dodecyl sulfate, and the supernatant was collected. A TIANamp Stool DNA Kit (Tiangen Biotech Co., Ltd., Beijing, China) was used to extract genomic DNA according to the manufacturer’s protocol. Before each extraction, the equipment was sterilized under ultraviolet (UV) light to prevent contamination from foreign DNA and PCR products^29^.

**Table 1:** Sample origin information.

Origin	Sample ID					
Hubei	yms1	yms2	yms3	yms4		
Heilongjiang	yms5	yms6				
Jiangxi	yms7	yms8				
Jilin	yms9	yms10	yms11			
Lilaoning	yms12	yms13				
Sichuan	yms14	yms15	yms16			
Shangdong	yms17					
Shanxi	yms18					
Yunnan	yms19	yms20				
Zhejiang	yms21	yms22	yms23	yms24	yms25	yms26

### PCR and high-throughput sequencing

The primers used in this study were an existing detection system^46^ based on the mitochondrial COI sequence. Metabarcoding studies on bulk collections of animals usually target a region within the COI “Folmer” region^30,47,48^. Therefore, the forward primer we used was LCO1490 (5′-GGTCAACAAATCATAAAGATATTGG-3′), and the reverse primer was HCO1777 (5′-ACTTATATTGTTTATACGAGGGAA-3′)^46^. Both primers were tagged with 8 bp tags at the 5' end to distinguish samples during data analysis (Table S3), and the final amplified product was 297 bp. PCR amplification was performed using Tks Gflex DNA Polymerase (Takara Bio Inc.). These PCRs were conducted in a 50 µl reaction volume containing 25 µl of 2× Gflex buffer, 2 µl of DNA, 1 µl each of the forward and reverse primers, 20 µl of ddH_2_O, and 1 µl of Tks Gflex DNA Polymerase. The PCR conditions consisted of an initial denaturation step at 98 ℃ for 1 min, followed by 40 cycles of denaturation at 98 ℃ for 10 s, annealing at 45 ℃ for 15 s, and extension at 68 ℃ for 30 s, with a final extension step at 68 ℃ for 5 min. To avoid contamination, all PCR steps were conducted in a sterile laminar flow hood that was physically separated from locations where DNA extraction or post-PCR sample processing occurred. The laminar flow hood was treated with UV light for 30 min prior to PCR preparation. We included negative controls at the DNA extraction and PCR steps. PCR products were visualized with 1% agarose gel electrophoresis. The PCR product (297 bp) was selected by excision on agarose gel, and non-specific PCR products and primer dimers were discarded. Subsequently, PCR products were mixed in equimolar amounts. A sequencing library was generated using an NEBNext^®^ Ultra™ DNA Library Prep Kit for Illumina (NEB, USA) following the manufacturer’s recommendations. The Illumina adapter sequences were 5′-AATGATACGGCGACCACCGAGATCTACACATCGGAGATCTTTCCCTACACGACGCTCTTCCGATCT-3′ and 5′-GATCGGAAGAGCACACGTCTGAACTCCAGTCACTCGACATCATCTCGTATGCCGTCTTCTGCTTG-3′. Sequencing was performed on the Illumina NovaSeq platform, and the read length was 2 × 250 bp.

### Bioinformatics processing

After quality control by FastQC (www.bioinformatics.babraham.ac.uk/projects/fastqc/), the sequencing reads were trimmed using Trimmomatic^49^, a trimming tool for Illumina sequencing data, to filter out the contaminating adapter sequence and low-quality reads (phred quality < 20). Sequencing reads were demultiplexed using fastq-multx^50^ and assigned to each sample according to the unique tags. Primer and tag sequences were trimmed using bbduk from BBMap tools^51^. Paired-end reads were merged using QIIME^52^. We dereplicated reads using the USEARCH^53–55^ “fastx_uniques” algorithm with the option “minuniquesize 12”. Then, we applied the USEARCH UNOISE3^36^ algorithm to detect and remove chimeras, substitutions due to incorrect base calls and gaps due to omitted or spurious base calls. USEARCH was used to cluster OTUs at a 98% similarity^35^ threshold. Finally, OTUs with a relative abundance less than 0.01% of total reads of the 26 samples were removed from the OTU table using QIIME (filter_otus_from_otu_table.py). A representative sequence from each OTU was then picked.

### Taxonomic assignment

We imported the OTU representative sequences into Geneious Prime 2020.2 and translated them into their amino acid sequences. After translation, we removed the sequences containing stop codons, which were regarded as artificial sequences. Then, the OTU table and representative sequences were regenerated. BLASTN^56^ was used to compare the OTU representative sequences against the NCBI GENBANK database, and the output was imported into MEGAN COMMUNITY EDITION version 6.10.8^57^. The representative sequences were filtered with identity values of 80% to remove non-metazoans. Order-level taxonomy was assigned at > 95% identity values, family-level taxonomy was assigned at > 96.5%, and species-level taxonomy was assigned when the identity values between the query and reference sequences were above 98%^31,35^. Finally, we manually checked the taxonomic assignment and investigated the distribution and life history of the identified species.

### Statistical analysis

We respectively rarefied (QIIME script-single_rarefaction.py^52^) the number of sequences per sample in the OTU table according to the sample with the smallest reads of bats and other biological compositions in their faeces to ensure comparability. Then, biodiversity analysis was performed after removing the host and non-metazoan sequences. The default parameters of R (v2.12.1) and the R package vegan (v2.0-1)^58^ were used for PCA, NMDS analysis and data visualization. The default parameters of the R packages phyloseq (v1.36.0)^59^ and ggplot2 (v3.3.3)^60^ were used for alpha diversity analysis and data visualization. Heatmap was plotted using the R package pheatmap (v1.0.12)^61^. We calculated the relative abundance (i.e., the value of each taxon divided by the total reads per sample) and the average relative abundance (i.e., the total relative abundance of each taxon across all samples divided by 26) for sample composition analyses^62^.

## Supplementary Information

Supplementary Information.

## Data Availability

Illumina data sets have been deposited in the Sequence Read Archive (SRA) under BioProject PRJNA688101.
