# Supplementary material for: Biological composition analysis of a natural medicine, Faeces Vespertilionis, with complex sources using DNA metabarcoding
Source: Sci Rep. 2022 Jan 10;12:375. doi: 10.1038/s41598-021-04387-1 (PMC8748881; doi:10.1038/s41598-021-04387-1)
Supplement: Supplementary file 1 — Supplementary Information. [file 41598_2021_4387_MOESM1_ESM.zip › Supplementary Material/Table S3.docx]

**Table S3.** Tags of primers corresponding to each sample.

| Sample ID | Forward primer tags | Reverse primer tags |
| --- | --- | --- |
| yms1 | TATACGCG | TATACGCG |
| yms2 | TATCTCGC | TATCTCGC |
| yms3 | TACTGCTG | TACTGCTG |
| yms4 | TACGAGAC | TACGAGAC |
| yms5 | TAGCGTCT | TAGCGTCT |
| yms6 | TCTGCATC | TCTGCATC |
| yms7 | TCATGTGC | TCATGTGC |
| yms8 | TCACTACG | TCACTACG |
| yms9 | TCGTAGCA | TCGTAGCA |
| yms10 | TGAGACGT | TGAGACGT |
| yms11 | TGCTCACT | TGCTCACT |
| yms12 | ATACGCTC | ATACGCTC |
| yms13 | ATAGAGCG | ATAGAGCG |
| yms14 | ATCACTGC | ATCACTGC |
| yms15 | ACTAGCAG | ACTAGCAG |
| yms16 | ACGATGTC | ACGATGTC |
| yms17 | ACGCGAGA | ACGCGAGA |
| yms18 | AGTCTGCA | AGTCTGCA |
| yms19 | AGATCGAC | AGATCGAC |
| yms20 | AGCGTATG | AGCGTATG |
| yms21 | CTAGTAGC | CTAGTAGC |
| yms22 | CTCTACGA | CTCTACGA |
| yms23 | CTCATGCT | CTCATGCT |
| yms24 | CTGTCTCG | CTGTCTCG |
| yms25 | CATGCTGT | CATGCTGT |
| yms26 | CAGCTGAG | CAGCTGAG |
