# Supplementary figures and images for: Biological composition analysis of a natural medicine, Faeces Vespertilionis, with complex sources using DNA metabarcoding
Source: Sci Rep. 2022 Jan 10;12:375. doi: 10.1038/s41598-021-04387-1 (PMC8748881; doi:10.1038/s41598-021-04387-1)

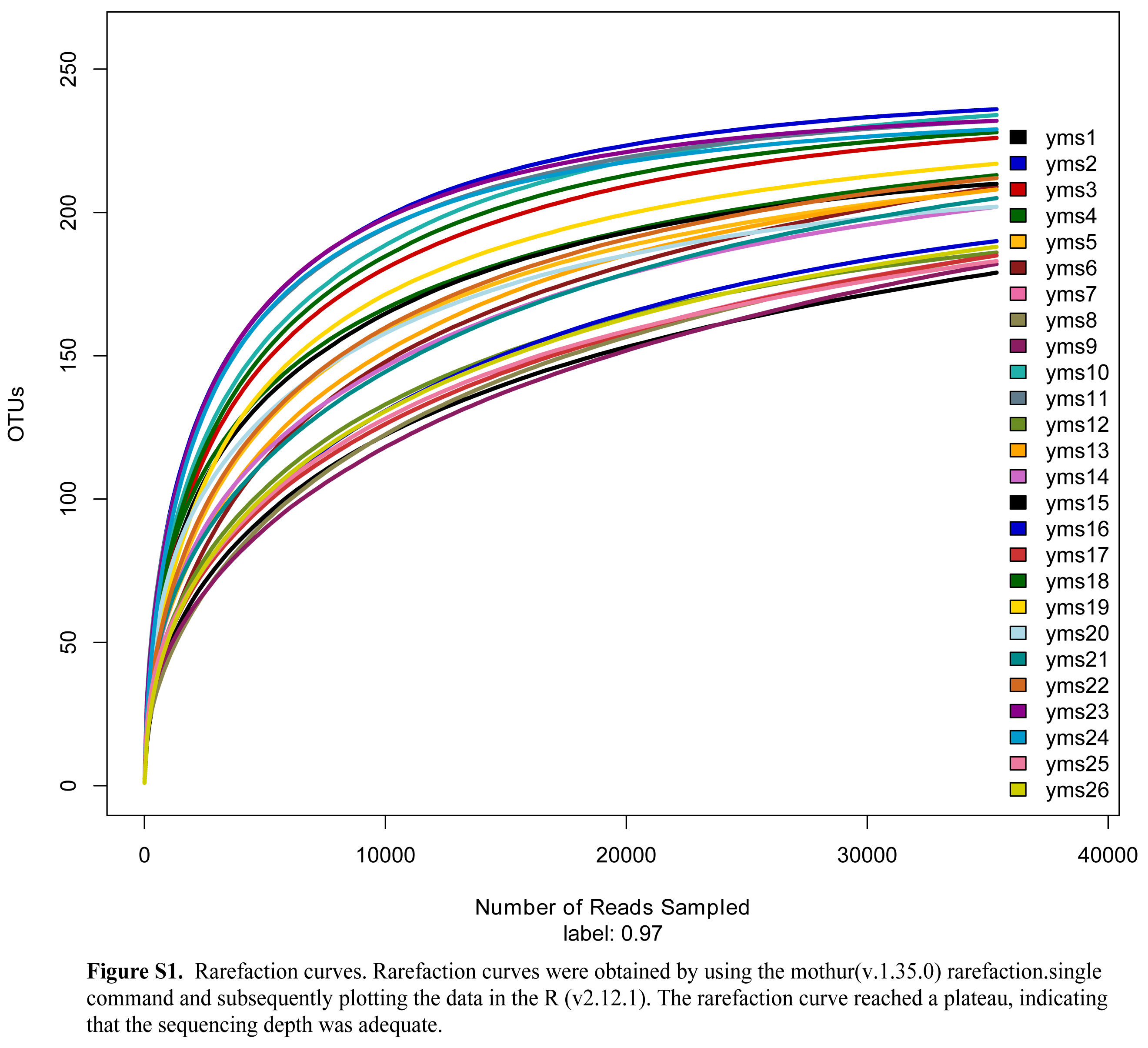

Supplement: Supplementary file 1 — Supplementary Information. [file 41598_2021_4387_MOESM1_ESM.zip › Supplementary Material/Figure S1.tif]

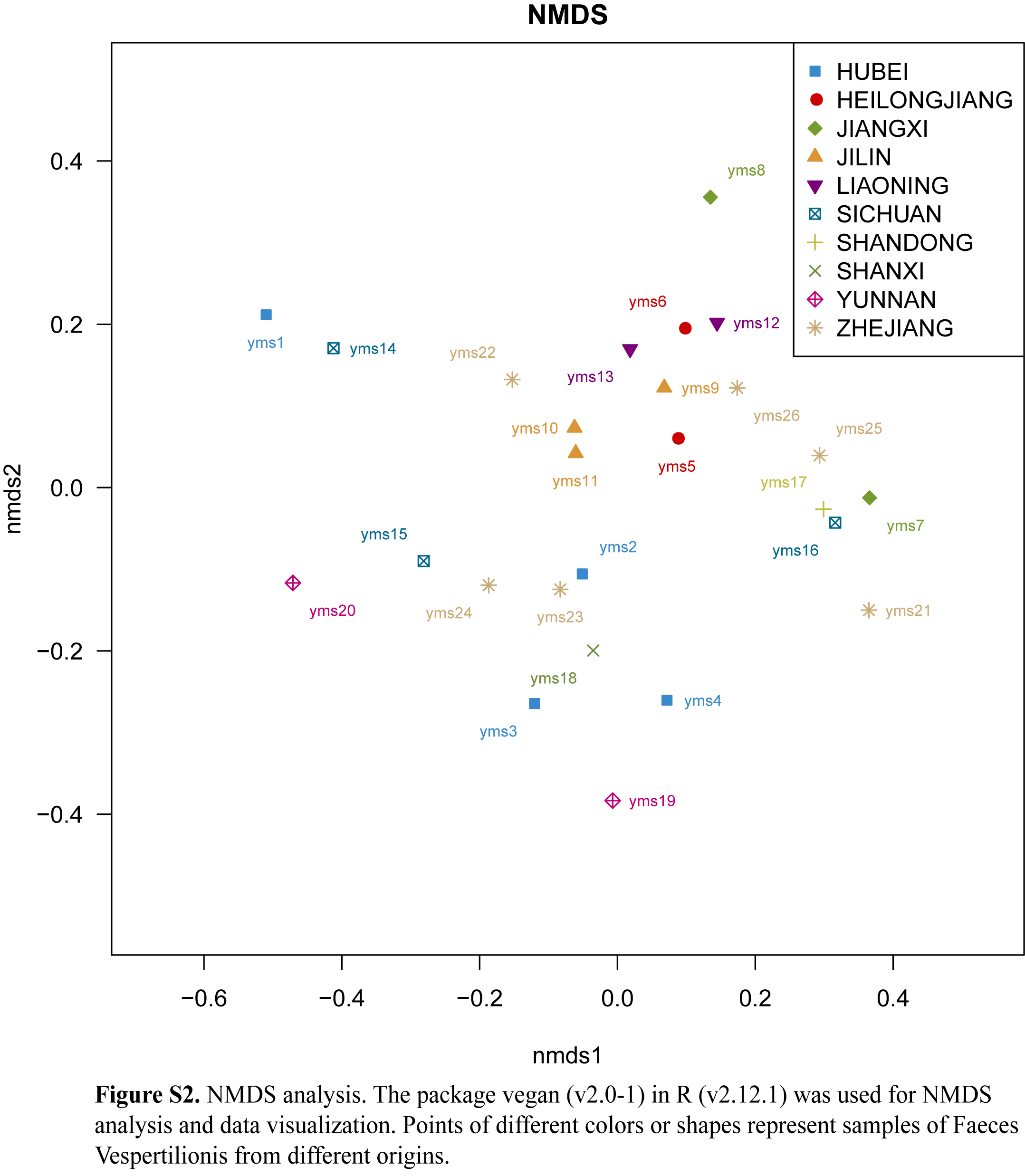

Supplement: Supplementary file 1 — Supplementary Information. [file 41598_2021_4387_MOESM1_ESM.zip › Supplementary Material/Figure S2.tif]
